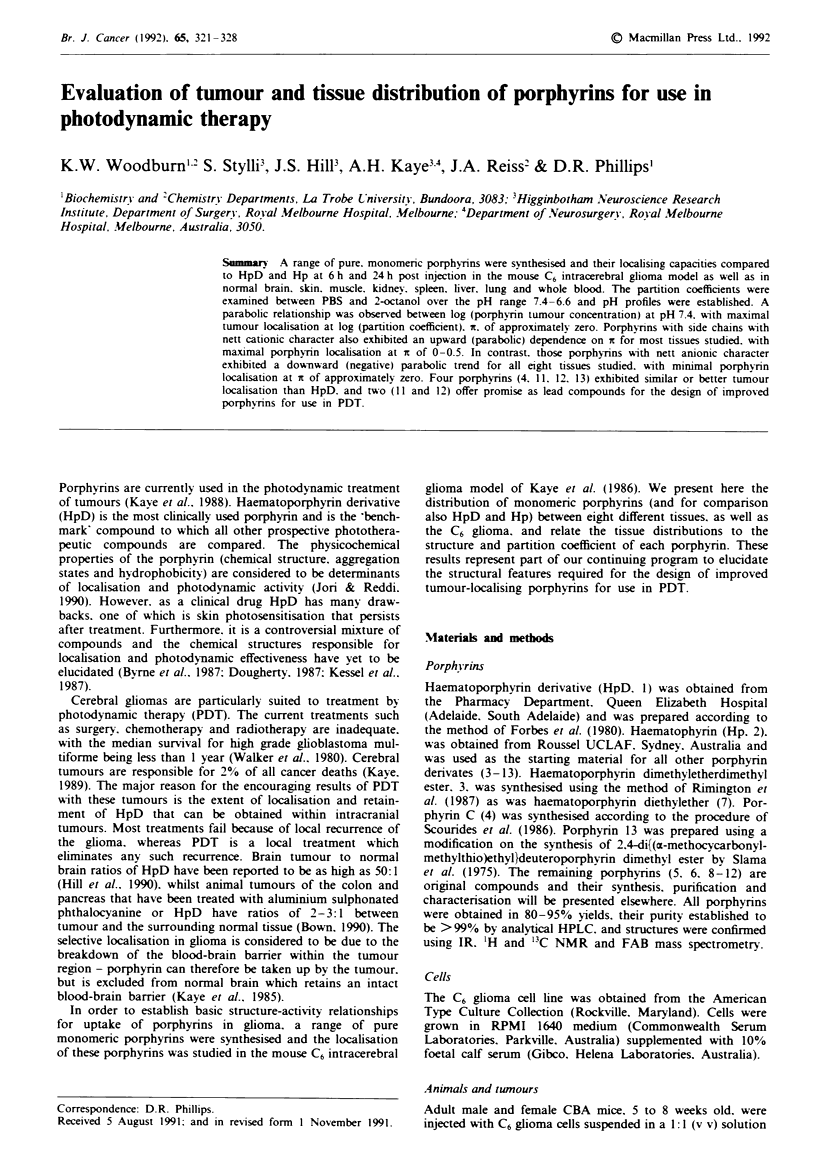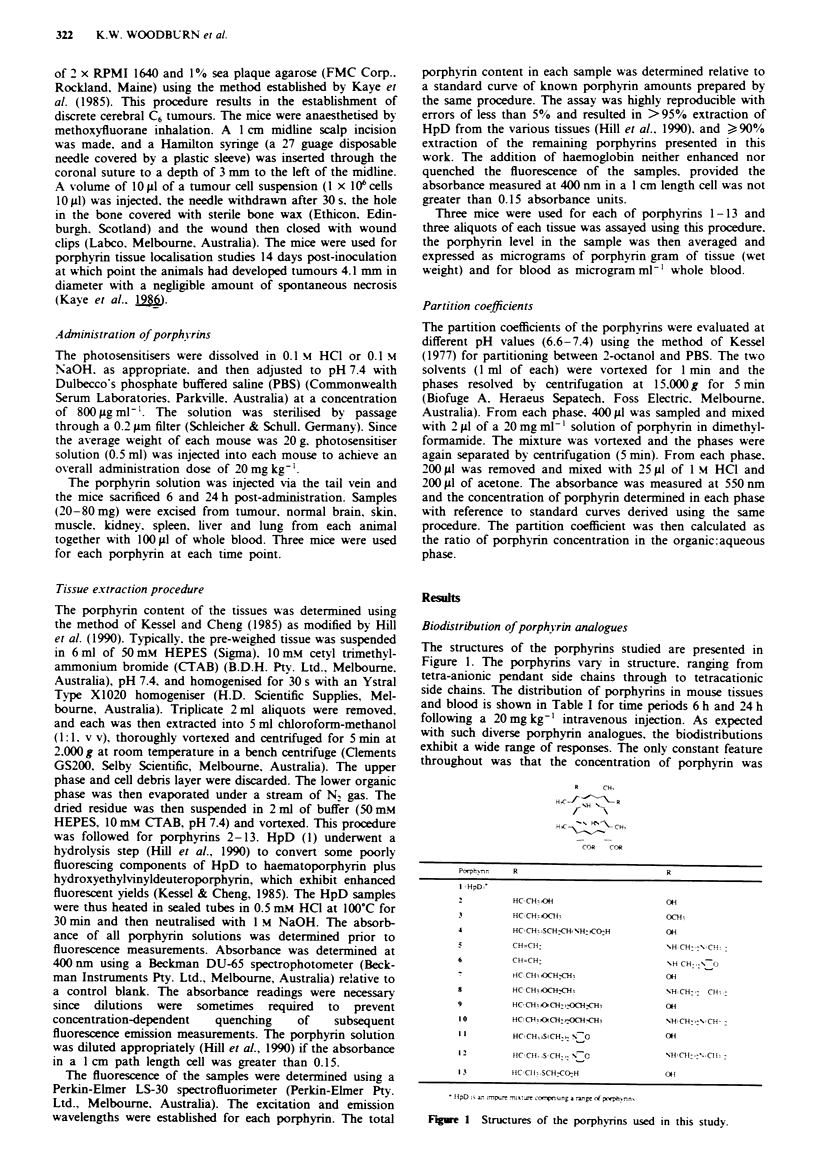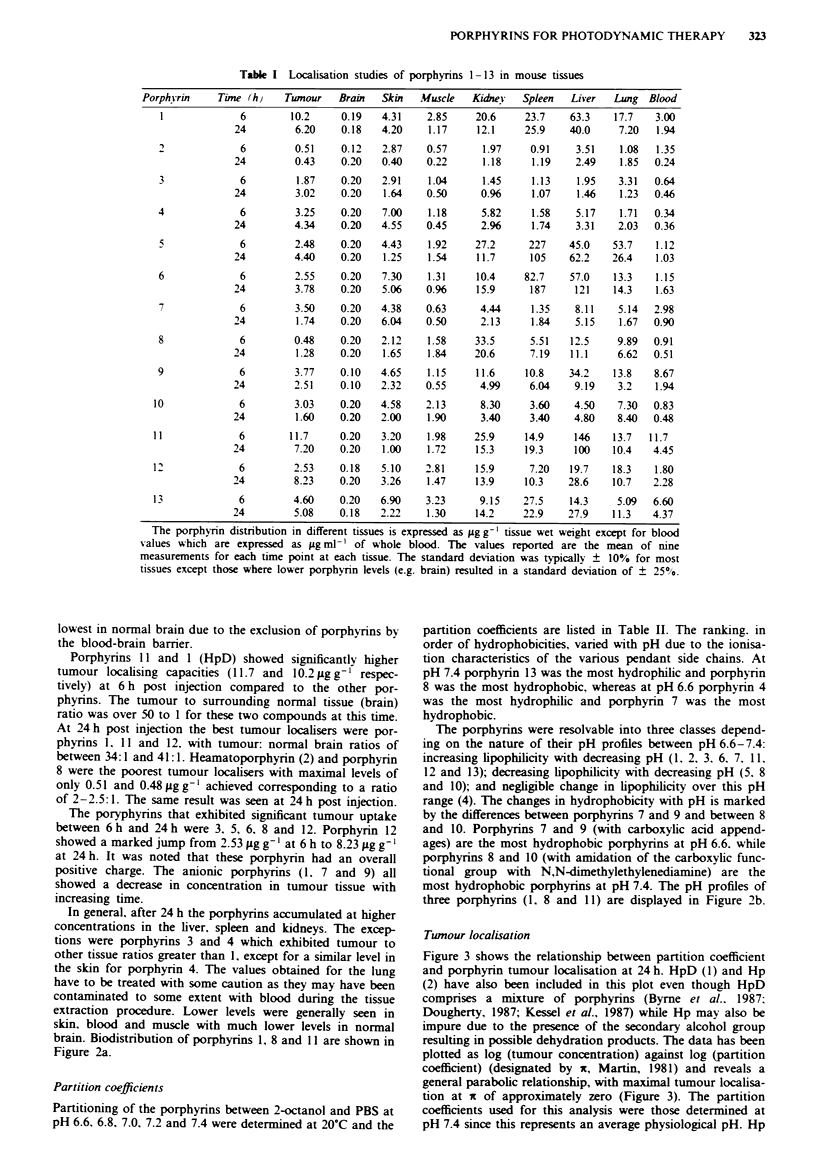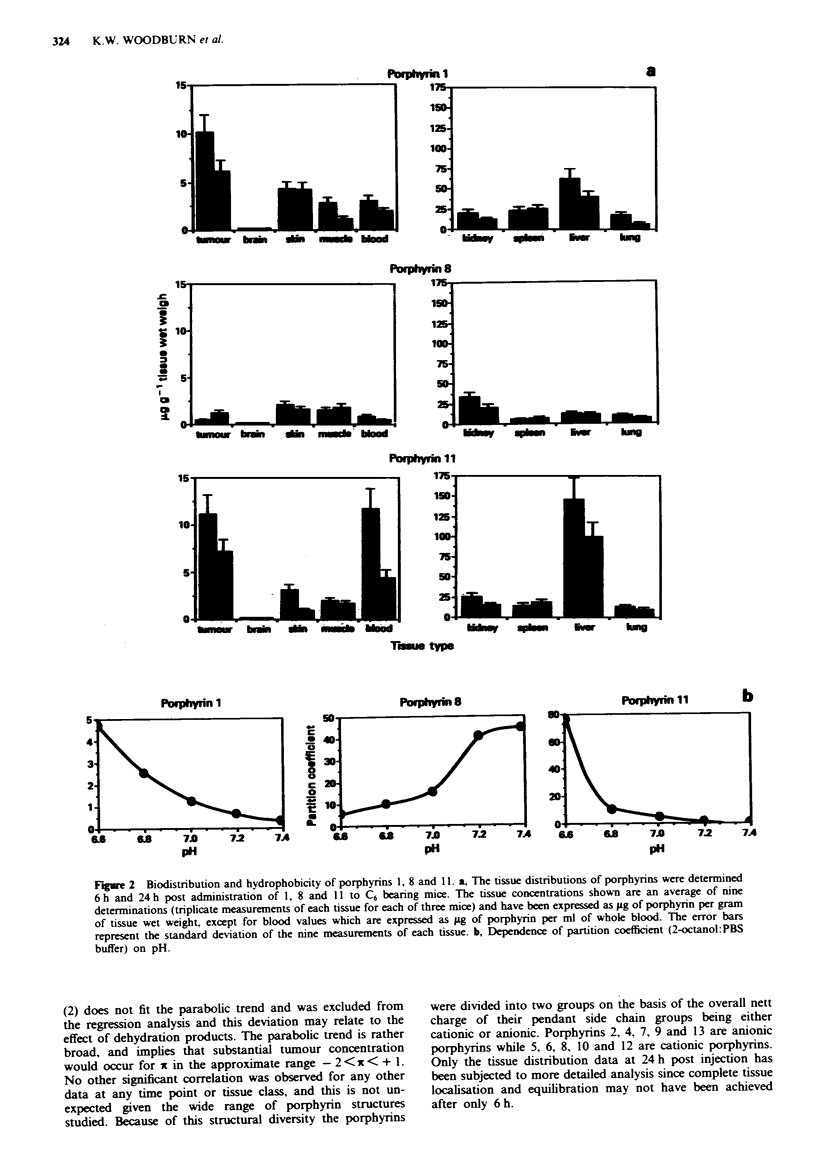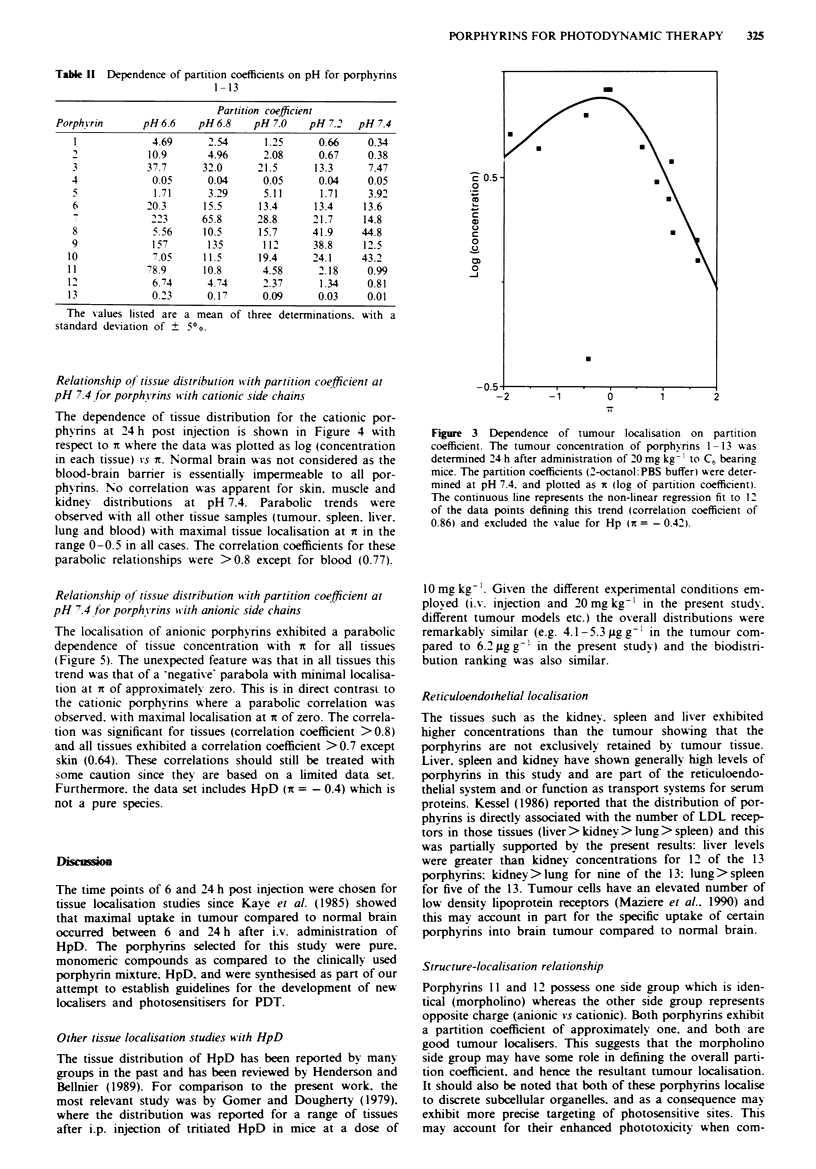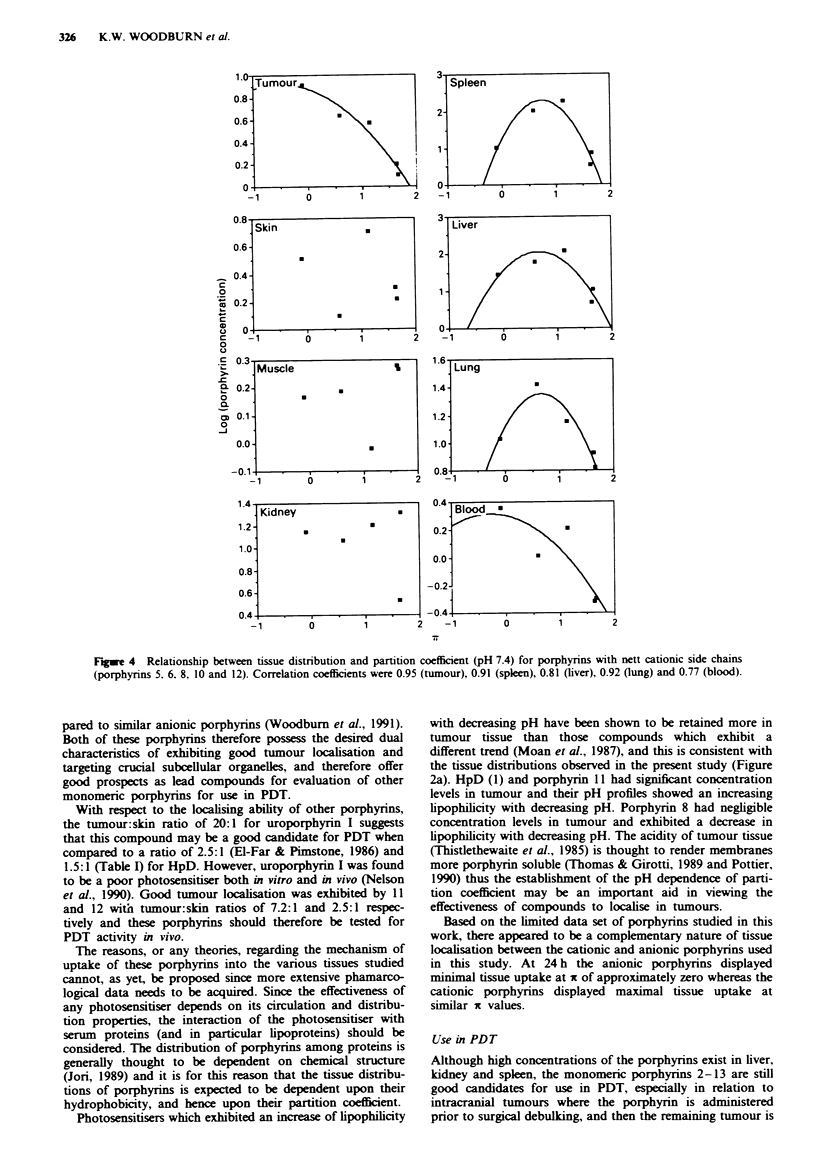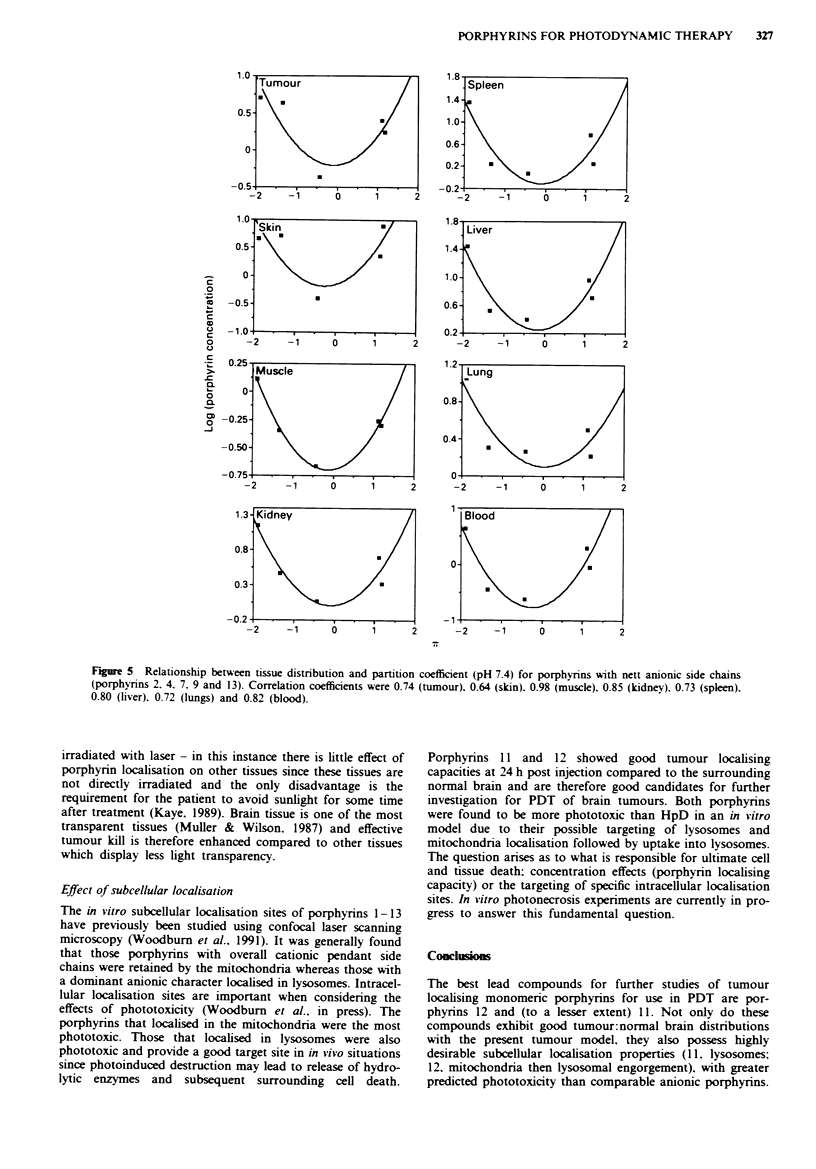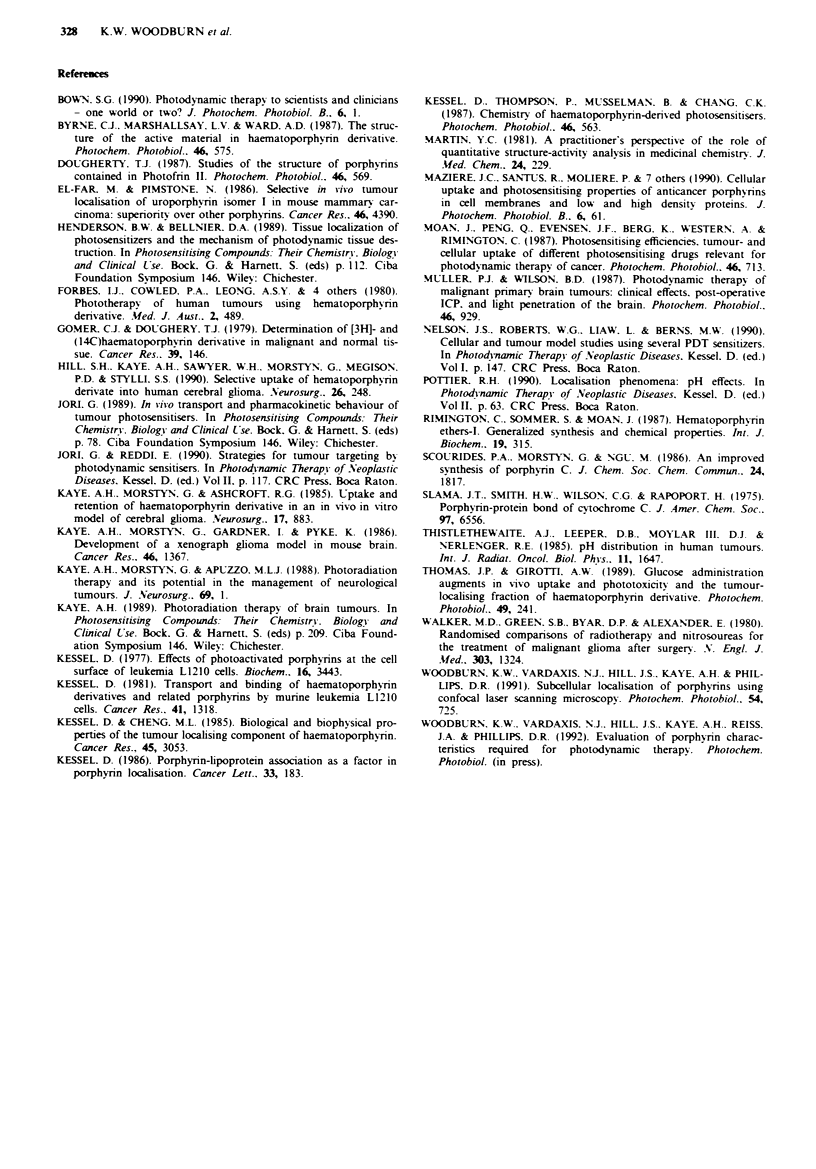# Evaluation of tumour and tissue distribution of porphyrins for use in photodynamic therapy.

**DOI:** 10.1038/bjc.1992.66

**Published:** 1992-03

**Authors:** K. W. Woodburn, S. Stylli, J. S. Hill, A. H. Kaye, J. A. Reiss, D. R. Phillips

**Affiliations:** Biochemistry Department, La Trobe University, Bundoora, Melbourne, Australia.

## Abstract

A range of pure, monomeric porphyrins were synthesised and their localising capacities compared to HpD and Hp at 6 h and 24 h post injection in the mouse C6 intracerebral glioma model as well as in normal brain, skin, muscle, kidney, spleen, liver, lung and whole blood. The partition coefficients were examined between PBS and 2-octanol over the pH range 7.4-6.6 and pH profiles were established. A parabolic relationship was observed between log (porphyrin tumour concentration) at pH 7.4, with maximal tumour localisation at log (partition coefficient), pi, of approximately zero. Porphyrins with side chains with nett cationic character also exhibited up upward (parabolic) dependence on pi for most tissues studied, with maximal porphyrin localisation at pi of 0-0.5. In contrast, those porphyrins with nett anionic character exhibited a downward (negative) parabolic trend for all eight tissues studied, with minimal porphyrin localisation at pi of approximately zero. Four porphyrins (4, 11, 12, 13) exhibited similar or better tumour localisation than HpD, and two (11 and 12) offer promise as lead compounds for the design of improved porphyrins for use in PDT.


Br. J. Cancer (1992), 65, 321-328 © Macmillan Press Ltd., 1992

### Evaluation of tumour and tissue distribution of porphyrins for use in photodynamic therapy

K.W. Woodburn[1,2] S. Stylli[3], J.S. Hill[3], A.H. Kaye[3,4], J.A. Reiss[2] & D.R. Phillips[1]

[1]*Biochemistry and* [2]*Chemistry Departments, La Trobe University, Bundoora, 3083;* [3]*Higginbotham Neuroscience Research Institute, Department of Surgery, Royal Melbourne Hospital, Melbourne;* [4]*Department of Neurosurgery, Royal Melbourne Hospital, Melbourne, Australia, 3050.*

**Summary** A range of pure, monomeric porphyrins were synthesised and their localising capacities compared to HpD and Hp at 6 h and 24 h post injection in the mouse $C_6$ intracerebral glioma model as well as in normal brain, skin, muscle, kidney, spleen, liver, lung and whole blood. The partition coefficients were examined between PBS and 2-octanol over the pH range 7.4-6.6 and pH profiles were established. A parabolic relationship was observed between log (porphyrin tumour concentration) at pH 7.4, with maximal tumour localisation at log (partition coefficient), $\pi$, of approximately zero. Porphyrins with side chains with nett cationic character also exhibited an upward (parabolic) dependence on $\pi$ for most tissues studied, with maximal porphyrin localisation at $\pi$ of 0-0.5. In contrast, those porphyrins with nett anionic character exhibited a downward (negative) parabolic trend for all eight tissues studied, with minimal porphyrin localisation at $\pi$ of approximately zero. Four porphyrins (4, 11, 12, 13) exhibited similar or better tumour localisation than HpD, and two (11 and 12) offer promise as lead compounds for the design of improved porphyrins for use in PDT.

Porphyrins are currently used in the photodynamic treatment of tumours (Kaye *et al.*, 1988). Haematoporphyrin derivative (HpD) is the most clinically used porphyrin and is the 'benchmark' compound to which all other prospective phototherapeutic compounds are compared. The physicochemical properties of the porphyrin (chemical structure, aggregation states and hydrophobicity) are considered to be determinants of localisation and photodynamic activity (Jori & Reddi, 1990). However, as a clinical drug HpD has many drawbacks, one of which is skin photosensitisation that persists after treatment. Furthermore, it is a controversial mixture of compounds and the chemical structures responsible for localisation and photodynamic effectiveness have yet to be elucidated (Byrne *et al.*, 1987; Dougherty, 1987; Kessel *et al.*, 1987).

Cerebral gliomas are particularly suited to treatment by photodynamic therapy (PDT). The current treatments such as surgery, chemotherapy and radiotherapy are inadequate, with the median survival for high grade glioblastoma multiforme being less than 1 year (Walker *et al.*, 1980). Cerebral tumours are responsible for 2% of all cancer deaths (Kaye, 1989). The major reason for the encouraging results of PDT with these tumours is the extent of localisation and retainment of HpD that can be obtained within intracranial tumours. Most treatments fail because of local recurrence of the glioma, whereas PDT is a local treatment which eliminates any such recurrence. Brain tumour to normal brain ratios of HpD have been reported to be as high as 50:1 (Hill *et al.*, 1990), whilst animal tumours of the colon and pancreas that have been treated with aluminium sulphonated phthalocyanine or HpD have ratios of 2-3:1 between tumour and the surrounding normal tissue (Bown, 1990). The selective localisation in glioma is considered to be due to the breakdown of the blood-brain barrier within the tumour region – porphyrin can therefore be taken up by the tumour, but is excluded from normal brain which retains an intact blood-brain barrier (Kaye *et al.*, 1985).

In order to establish basic structure-activity relationships for uptake of porphyrins in glioma, a range of pure monomeric porphyrins were synthesised and the localisation of these porphyrins was studied in the mouse $C_6$ intracerebral glioma model of Kaye *et al.* (1986). We present here the distribution of monomeric porphyrins (and for comparison also HpD and Hp) between eight different tissues, as well as the $C_6$ glioma, and relate the tissue distributions to the structure and partition coefficient of each porphyrin. These results represent part of our continuing program to elucidate the structural features required for the design of improved tumour-localising porphyrins for use in PDT.

Correspondence: D.R. Phillips.
Received 5 August 1991; and in revised form 1 November 1991.

### Materials and methods

### *Porphyrins*

Haematoporphyrin derivative (HpD, 1) was obtained from the Pharmacy Department, Queen Elizabeth Hospital (Adelaide, South Adelaide) and was prepared according to the method of Forbes *et al.* (1980). Haematophyrin (Hp, 2), was obtained from Roussel UCLAF, Sydney, Australia and was used as the starting material for all other porphyrin derivates (3-13). Haematoporphyrin dimethyletherdimethyl ester, 3, was synthesised using the method of Rimington *et al.* (1987) as was haematoporphyrin diethylether (7). Porphyrin C (4) was synthesised according to the procedure of Scourides *et al.* (1986). Porphyrin 13 was prepared using a modification on the synthesis of 2,4-di{(α-methocycarbonylmethylthio)ethyl}deuteroporphyrin dimethyl ester by Slama *et al.* (1975). The remaining porphyrins (5, 6, 8-12) are original compounds and their synthesis, purification and characterisation will be presented elsewhere. All porphyrins were obtained in 80-95% yields, their purity established to be >99% by analytical HPLC, and structures were confirmed using IR, $^1H$ and $^{13}C$ NMR and FAB mass spectrometry.

### *Cells*

The $C_6$ glioma cell line was obtained from the American Type Culture Collection (Rockville, Maryland). Cells were grown in RPMI 1640 medium (Commonwealth Serum Laboratories, Parkville, Australia) supplemented with 10% foetal calf serum (Gibco, Helena Laboratories, Australia).

### *Animals and tumours*

Adult male and female CBA mice, 5 to 8 weeks old, were injected with $C_6$ glioma cells suspended in a 1:1 (v v) solution of 2 × RPMI 1640 and 1% sea plaque agarose (FMC Corp., Rockland, Maine) using the method established by Kaye et al. (1985). This procedure results in the establishment of discrete cerebral $C_6$ tumours. The mice were anaesthetised by methoxyfluorane inhalation. A 1 cm midline scalp incision was made, and a Hamilton syringe (a 27 guage disposable needle covered by a plastic sleeve) was inserted through the coronal suture to a depth of 3 mm to the left of the midline. A volume of 10 µl of a tumour cell suspension (1 × $10^6$ cells 10 µl) was injected, the needle withdrawn after 30 s, the hole in the bone covered with sterile bone wax (Ethicon, Edinburgh, Scotland) and the wound then closed with wound clips (Labco, Melbourne, Australia). The mice were used for porphyrin tissue localisation studies 14 days post-inoculation at which point the animals had developed tumours 4.1 mm in diameter with a negligible amount of spontaneous necrosis (Kaye et al., 1986).

### *Administration of porphyrins*

The photosensitisers were dissolved in 0.1 M HCl or 0.1 M NaOH, as appropriate, and then adjusted to pH 7.4 with Dulbecco's phosphate buffered saline (PBS) (Commonwealth Serum Laboratories, Parkville, Australia) at a concentration of 800 µg $ml^{-1}$. The solution was sterilised by passage through a 0.2 µm filter (Schleicher & Schull, Germany). Since the average weight of each mouse was 20 g, photosensitiser solution (0.5 ml) was injected into each mouse to achieve an overall administration dose of 20 mg $kg^{-1}$.

The porphyrin solution was injected via the tail vein and the mice sacrificed 6 and 24 h post-administration. Samples (20–80 mg) were excised from tumour, normal brain, skin, muscle, kidney, spleen, liver and lung from each animal together with 100 µl of whole blood. Three mice were used for each porphyrin at each time point.

### *Tissue extraction procedure*

The porphyrin content of the tissues was determined using the method of Kessel and Cheng (1985) as modified by Hill et al. (1990). Typically, the pre-weighed tissue was suspended in 6 ml of 50 mM HEPES (Sigma), 10 mM cetyl trimethylammonium bromide (CTAB) (B.D.H. Pty. Ltd., Melbourne, Australia), pH 7.4, and homogenised for 30 s with an Ystral Type X1020 homogeniser (H.D. Scientific Supplies, Melbourne, Australia). Triplicate 2 ml aliquots were removed, and each was then extracted into 5 ml chloroform-methanol (1:1, v v), thoroughly vortexed and centrifuged for 5 min at 2,000 g at room temperature in a bench centrifuge (Clements GS200, Selby Scientific, Melbourne, Australia). The upper phase and cell debris layer were discarded. The lower organic phase was then evaporated under a stream of $N_2$ gas. The dried residue was then suspended in 2 ml of buffer (50 mM HEPES, 10 mM CTAB, pH 7.4) and vortexed. This procedure was followed for porphyrins 2–13. HpD (1) underwent a hydrolysis step (Hill et al., 1990) to convert some poorly fluorescing components of HpD to haematoporphyrin plus hydroxyethylvinyldeuteroporphyrin, which exhibit enhanced fluorescent yields (Kessel & Cheng, 1985). The HpD samples were thus heated in sealed tubes in 0.5 mM HCl at 100°C for 30 min and then neutralised with 1 M NaOH. The absorbance of all porphyrin solutions was determined prior to fluorescence measurements. Absorbance was determined at 400 nm using a Beckman DU-65 spectrophotometer (Beckman Instruments Pty. Ltd., Melbourne, Australia) relative to a control blank. The absorbance readings were necessary since dilutions were sometimes required to prevent concentration-dependent quenching of subsequent fluorescence emission measurements. The porphyrin solution was diluted appropriately (Hill et al., 1990) if the absorbance in a 1 cm path length cell was greater than 0.15.

The fluorescence of the samples were determined using a Perkin-Elmer LS-30 spectrofluorimeter (Perkin-Elmer Pty. Ltd., Melbourne, Australia). The excitation and emission wavelengths were established for each porphyrin. The total porphyrin content in each sample was determined relative to a standard curve of known porphyrin amounts prepared by the same procedure. The assay was highly reproducible with errors of less than 5% and resulted in >95% extraction of HpD from the various tissues (Hill et al., 1990), and ⩾90% extraction of the remaining porphyrins presented in this work. The addition of haemoglobin neither enhanced nor quenched the fluorescence of the samples, provided the absorbance measured at 400 nm in a 1 cm length cell was not greater than 0.15 absorbance units.

Three mice were used for each of porphyrins 1–13 and three aliquots of each tissue was assayed using this procedure, the porphyrin level in the sample was then averaged and expressed as micrograms of porphyrin gram of tissue (wet weight) and for blood as microgram $ml^{-1}$ whole blood.

### *Partition coefficients*

The partition coefficients of the porphyrins were evaluated at different pH values (6.6–7.4) using the method of Kessel (1977) for partitioning between 2-octanol and PBS. The two solvents (1 ml of each) were vortexed for 1 min and the phases resolved by centrifugation at 15,000 g for 5 min (Biofuge A, Heraeus Sepatech, Foss Electric, Melbourne, Australia). From each phase, 400 µl was sampled and mixed with 2 µl of a 20 mg $ml^{-1}$ solution of porphyrin in dimethylformamide. The mixture was vortexed and the phases were again separated by centrifugation (5 min). From each phase, 200 µl was removed and mixed with 25 µl of 1 M HCl and 200 µl of acetone. The absorbance was measured at 550 nm and the concentration of porphyrin determined in each phase with reference to standard curves derived using the same procedure. The partition coefficient was then calculated as the ratio of porphyrin concentration in the organic:aqueous phase.

### Results

### *Biodistribution of porphyrin analogues*

The structures of the porphyrins studied are presented in Figure 1. The porphyrins vary in structure, ranging from tetra-anionic pendant side chains through to tetracationic side chains. The distribution of porphyrins in mouse tissues and blood is shown in Table I for time periods 6 h and 24 h following a 20 mg $kg^{-1}$ intravenous injection. As expected with such diverse porphyrin analogues, the biodistributions exhibit a wide range of responses. The only constant feature throughout was that the concentration of porphyrin was

| Porphyrin | R | R |
|---|---|---|
| 1 (HpD)* | | |
| 2 | $HC(CH_3)OH$ | OH |
| 3 | $HC(CH_3)OCH_3$ | $OCH_3$ |
| 4 | $HC(CH_3)SCH_2CH(NH_2)CO_2H$ | OH |
| 5 | $CH=CH_2$ | $NH(CH_2)_2N(CH_3)_2$ |
| 6 | $CH=CH_2$ | $NH(CH_2)_2N$⌒O |
| 7 | $HC(CH_3)OCH_2CH_3$ | OH |
| 8 | $HC(CH_3)OCH_2CH_3$ | $NH(CH_2)_2$ [illegible] $CH_3)_2$ |
| 9 | $HC(CH_3)O(CH_2)_2OCH_2CH_3$ | OH |
| 10 | $HC(CH_3)O(CH_2)_2OCH_2CH_3$ | $NH(CH_2)_2N(CH_3)_2$ |
| 11 | $HC(CH_3)S(CH_2)_2$ N⌒O | OH |
| 12 | $HC(CH_3)S(CH_2)_2$ N⌒O | $NH(CH_2)_2N(CH_3)_2$ |
| 13 | $HC(CH_3)SCH_2CO_2H$ | OH |

* HpD is an impure mixture comprising a range of porphyrins

**Figure 1** Structures of the porphyrins used in this study.

**Table I** Localisation studies of porphyrins 1–13 in mouse tissues

| *Porphyrin* | *Time (h)* | *Tumour* | *Brain* | *Skin* | *Muscle* | *Kidney* | *Spleen* | *Liver* | *Lung* | *Blood* |
|---|---|---|---|---|---|---|---|---|---|---|
| 1 | 6 | 10.2 | 0.19 | 4.31 | 2.85 | 20.6 | 23.7 | 63.3 | 17.7 | 3.00 |
| | 24 | 6.20 | 0.18 | 4.20 | 1.17 | 12.1 | 25.9 | 40.0 | 7.20 | 1.94 |
| 2 | 6 | 0.51 | 0.12 | 2.87 | 0.57 | 1.97 | 0.91 | 3.51 | 1.08 | 1.35 |
| | 24 | 0.43 | 0.20 | 0.40 | 0.22 | 1.18 | 1.19 | 2.49 | 1.85 | 0.24 |
| 3 | 6 | 1.87 | 0.20 | 2.91 | 1.04 | 1.45 | 1.13 | 1.95 | 3.31 | 0.64 |
| | 24 | 3.02 | 0.20 | 1.64 | 0.50 | 0.96 | 1.07 | 1.46 | 1.23 | 0.46 |
| 4 | 6 | 3.25 | 0.20 | 7.00 | 1.18 | 5.82 | 1.58 | 5.17 | 1.71 | 0.34 |
| | 24 | 4.34 | 0.20 | 4.55 | 0.45 | 2.96 | 1.74 | 3.31 | 2.03 | 0.36 |
| 5 | 6 | 2.48 | 0.20 | 4.43 | 1.92 | 27.2 | 227 | 45.0 | 53.7 | 1.12 |
| | 24 | 4.40 | 0.20 | 1.25 | 1.54 | 11.7 | 105 | 62.2 | 26.4 | 1.03 |
| 6 | 6 | 2.55 | 0.20 | 7.30 | 1.31 | 10.4 | 82.7 | 57.0 | 13.3 | 1.15 |
| | 24 | 3.78 | 0.20 | 5.06 | 0.96 | 15.9 | 187 | 121 | 14.3 | 1.63 |
| 7 | 6 | 3.50 | 0.20 | 4.38 | 0.63 | 4.44 | 1.35 | 8.11 | 5.14 | 2.98 |
| | 24 | 1.74 | 0.20 | 6.04 | 0.50 | 2.13 | 1.84 | 5.15 | 1.67 | 0.90 |
| 8 | 6 | 0.48 | 0.20 | 2.12 | 1.58 | 33.5 | 5.51 | 12.5 | 9.89 | 0.91 |
| | 24 | 1.28 | 0.20 | 1.65 | 1.84 | 20.6 | 7.19 | 11.1 | 6.62 | 0.51 |
| 9 | 6 | 3.77 | 0.10 | 4.65 | 1.15 | 11.6 | 10.8 | 34.2 | 13.8 | 8.67 |
| | 24 | 2.51 | 0.10 | 2.32 | 0.55 | 4.99 | 6.04 | 9.19 | 3.2 | 1.94 |
| 10 | 6 | 3.03 | 0.20 | 4.58 | 2.13 | 8.30 | 3.60 | 4.50 | 7.30 | 0.83 |
| | 24 | 1.60 | 0.20 | 2.00 | 1.90 | 3.40 | 3.40 | 4.80 | 8.40 | 0.48 |
| 11 | 6 | 11.7 | 0.20 | 3.20 | 1.98 | 25.9 | 14.9 | 146 | 13.7 | 11.7 |
| | 24 | 7.20 | 0.20 | 1.00 | 1.72 | 15.3 | 19.3 | 100 | 10.4 | 4.45 |
| 12 | 6 | 2.53 | 0.18 | 5.10 | 2.81 | 15.9 | 7.20 | 19.7 | 18.3 | 1.80 |
| | 24 | 8.23 | 0.20 | 3.26 | 1.47 | 13.9 | 10.3 | 28.6 | 10.7 | 2.28 |
| 13 | 6 | 4.60 | 0.20 | 6.90 | 3.23 | 9.15 | 27.5 | 14.3 | 5.09 | 6.60 |
| | 24 | 5.08 | 0.18 | 2.22 | 1.30 | 14.2 | 22.9 | 27.9 | 11.3 | 4.37 |

The porphyrin distribution in different tissues is expressed as $\mu g\ g^{-1}$ tissue wet weight except for blood values which are expressed as $\mu g\ ml^{-1}$ of whole blood. The values reported are the mean of nine measurements for each time point at each tissue. The standard deviation was typically ± 10% for most tissues except those where lower porphyrin levels (e.g. brain) resulted in a standard deviation of ± 25%.

lowest in normal brain due to the exclusion of porphyrins by the blood-brain barrier.

Porphyrins 11 and 1 (HpD) showed significantly higher tumour localising capacities (11.7 and 10.2 $\mu g\ g^{-1}$ respectively) at 6 h post injection compared to the other porphyrins. The tumour to surrounding normal tissue (brain) ratio was over 50 to 1 for these two compounds at this time. At 24 h post injection the best tumour localisers were porphyrins 1, 11 and 12, with tumour: normal brain ratios of between 34:1 and 41:1. Heamatoporphyrin (2) and porphyrin 8 were the poorest tumour localisers with maximal levels of only 0.51 and 0.48 $\mu g\ g^{-1}$ achieved corresponding to a ratio of 2–2.5:1. The same result was seen at 24 h post injection.

The poryphyrins that exhibited significant tumour uptake between 6 h and 24 h were 3, 5, 6, 8 and 12. Porphyrin 12 showed a marked jump from 2.53 $\mu g\ g^{-1}$ at 6 h to 8.23 $\mu g\ g^{-1}$ at 24 h. It was noted that these porphyrin had an overall positive charge. The anionic porphyrins (1, 7 and 9) all showed a decrease in concentration in tumour tissue with increasing time.

In general, after 24 h the porphyrins accumulated at higher concentrations in the liver, spleen and kidneys. The exceptions were porphyrins 3 and 4 which exhibited tumour to other tissue ratios greater than 1, except for a similar level in the skin for porphyrin 4. The values obtained for the lung have to be treated with some caution as they may have been contaminated to some extent with blood during the tissue extraction procedure. Lower levels were generally seen in skin, blood and muscle with much lower levels in normal brain. Biodistribution of porphyrins 1, 8 and 11 are shown in Figure 2a.

*Partition coefficients*

Partitioning of the porphyrins between 2-octanol and PBS at pH 6.6, 6.8, 7.0, 7.2 and 7.4 were determined at 20°C and the partition coefficients are listed in Table II. The ranking, in order of hydrophobicities, varied with pH due to the ionisation characteristics of the various pendant side chains. At pH 7.4 porphyrin 13 was the most hydrophilic and porphyrin 8 was the most hydrophobic, whereas at pH 6.6 porphyrin 4 was the most hydrophilic and porphyrin 7 was the most hydrophobic.

The porphyrins were resolvable into three classes depending on the nature of their pH profiles between pH 6.6–7.4: increasing lipophilicity with decreasing pH (1, 2, 3, 6, 7, 11, 12 and 13); decreasing lipophilicity with decreasing pH (5, 8 and 10); and negligible change in lipophilicity over this pH range (4). The changes in hydrophobicity with pH is marked by the differences between porphyrins 7 and 9 and between 8 and 10. Porphyrins 7 and 9 (with carboxylic acid appendages) are the most hydrophobic porphyrins at pH 6.6, while porphyrins 8 and 10 (with amidation of the carboxylic functional group with N,N-dimethylethylenediamine) are the most hydrophobic porphyrins at pH 7.4. The pH profiles of three porphyrins (1, 8 and 11) are displayed in Figure 2b.

*Tumour localisation*

Figure 3 shows the relationship between partition coefficient and porphyrin tumour localisation at 24 h. HpD (1) and Hp (2) have also been included in this plot even though HpD comprises a mixture of porphyrins (Byrne *et al.*, 1987; Dougherty, 1987; Kessel *et al.*, 1987) while Hp may also be impure due to the presence of the secondary alcohol group resulting in possible dehydration products. The data has been plotted as log (tumour concentration) against log (partition coefficient) (designated by $\pi$, Martin, 1981) and reveals a general parabolic relationship, with maximal tumour localisation at $\pi$ of approximately zero (Figure 3). The partition coefficients used for this analysis were those determined at pH 7.4 since this represents an average physiological pH. Hp

**Figure 2** Biodistribution and hydrophobicity of porphyrins 1, 8 and 11. **a**, The tissue distributions of porphyrins were determined 6 h and 24 h post administration of 1, 8 and 11 to $C_6$ bearing mice. The tissue concentrations shown are an average of nine determinations (triplicate measurements of each tissue for each of three mice) and have been expressed as μg of porphyrin per gram of tissue wet weight, except for blood values which are expressed as μg of porphyrin per ml of whole blood. The error bars represent the standard deviation of the nine measurements of each tissue. **b**, Dependence of partition coefficient (2-octanol:PBS buffer) on pH.

(2) does not fit the parabolic trend and was excluded from the regression analysis and this deviation may relate to the effect of dehydration products. The parabolic trend is rather broad, and implies that substantial tumour concentration would occur for $\pi$ in the approximate range $-2<\pi<+1$. No other significant correlation was observed for any other data at any time point or tissue class, and this is not unexpected given the wide range of porphyrin structures studied. Because of this structural diversity the porphyrins were divided into two groups on the basis of the overall nett charge of their pendant side chain groups being either cationic or anionic. Porphyrins 2, 4, 7, 9 and 13 are anionic porphyrins while 5, 6, 8, 10 and 12 are cationic porphyrins. Only the tissue distribution data at 24 h post injection has been subjected to more detailed analysis since complete tissue localisation and equilibration may not have been achieved after only 6 h.

**Table II** Dependence of partition coefficients on pH for porphyrins 1–13

| Porphyrin | Partition coefficient pH 6.6 | pH 6.8 | pH 7.0 | pH 7.2 | pH 7.4 |
|---|---|---|---|---|---|
| 1 | 4.69 | 2.54 | 1.25 | 0.66 | 0.34 |
| 2 | 10.9 | 4.96 | 2.08 | 0.67 | 0.38 |
| 3 | 37.7 | 32.0 | 21.5 | 13.3 | 7.47 |
| 4 | 0.05 | 0.04 | 0.05 | 0.04 | 0.05 |
| 5 | 1.71 | 3.29 | 5.11 | 1.71 | 3.92 |
| 6 | 20.3 | 15.5 | 13.4 | 13.4 | 13.6 |
| 7 | 223 | 65.8 | 28.8 | 21.7 | 14.8 |
| 8 | 5.56 | 10.5 | 15.7 | 41.9 | 44.8 |
| 9 | 157 | 135 | 112 | 38.8 | 12.5 |
| 10 | 7.05 | 11.5 | 19.4 | 24.1 | 43.2 |
| 11 | 78.9 | 10.8 | 4.58 | 2.18 | 0.99 |
| 12 | 6.74 | 4.74 | 2.37 | 1.34 | 0.81 |
| 13 | 0.23 | 0.17 | 0.09 | 0.03 | 0.01 |

The values listed are a mean of three determinations, with a standard deviation of ± 5%.

**Figure 3** Dependence of tumour localisation on partition coefficient. The tumour concentration of porphyrins 1–13 was determined 24 h after administration of 20 mg kg$^{-1}$ to $C_6$ bearing mice. The partition coefficients (2-octanol:PBS buffer) were determined at pH 7.4, and plotted as π (log of partition coefficient). The continuous line represents the non-linear regression fit to 12 of the data points defining this trend (correlation coefficient of 0.86) and excluded the value for Hp (π = – 0.42).

*Relationship of tissue distribution with partition coefficient at pH 7.4 for porphyrins with cationic side chains*

The dependence of tissue distribution for the cationic porphyrins at 24 h post injection is shown in Figure 4 with respect to π where the data was plotted as log (concentration in each tissue) *vs* π. Normal brain was not considered as the blood-brain barrier is essentially impermeable to all porphyrins. No correlation was apparent for skin, muscle and kidney distributions at pH 7.4. Parabolic trends were observed with all other tissue samples (tumour, spleen, liver, lung and blood) with maximal tissue localisation at π in the range 0–0.5 in all cases. The correlation coefficients for these parabolic relationships were >0.8 except for blood (0.77).

*Relationship of tissue distribution with partition coefficient at pH 7.4 for porphyrins with anionic side chains*

The localisation of anionic porphyrins exhibited a parabolic dependence of tissue concentration with π for all tissues (Figure 5). The unexpected feature was that in all tissues this trend was that of a 'negative' parabola with minimal localisation at π of approximately zero. This is in direct contrast to the cationic porphyrins where a parabolic correlation was observed, with maximal localisation at π of zero. The correlation was significant for tissues (correlation coefficient >0.8) and all tissues exhibited a correlation coefficient >0.7 except skin (0.64). These correlations should still be treated with some caution since they are based on a limited data set. Furthermore, the data set includes HpD (π = – 0.4) which is not a pure species.

### Discussion

The time points of 6 and 24 h post injection were chosen for tissue localisation studies since Kaye *et al.* (1985) showed that maximal uptake in tumour compared to normal brain occurred between 6 and 24 h after i.v. administration of HpD. The porphyrins selected for this study were pure, monomeric compounds as compared to the clinically used porphyrin mixture, HpD, and were synthesised as part of our attempt to establish guidelines for the development of new localisers and photosensitisers for PDT.

*Other tissue localisation studies with HpD*

The tissue distribution of HpD has been reported by many groups in the past and has been reviewed by Henderson and Bellnier (1989). For comparison to the present work, the most relevant study was by Gomer and Dougherty (1979), where the distribution was reported for a range of tissues after i.p. injection of tritiated HpD in mice at a dose of 10 mg kg$^{-1}$. Given the different experimental conditions employed (i.v. injection and 20 mg kg$^{-1}$ in the present study, different tumour models etc.) the overall distributions were remarkably similar (e.g. 4.1–5.3 μg g$^{-1}$ in the tumour compared to 6.2 μg g$^{-1}$ in the present study) and the biodistribution ranking was also similar.

*Reticuloendothelial localisation*

The tissues such as the kidney, spleen and liver exhibited higher concentrations than the tumour showing that the porphyrins are not exclusively retained by tumour tissue. Liver, spleen and kidney have shown generally high levels of porphyrins in this study and are part of the reticuloendothelial system and/or function as transport systems for serum proteins. Kessel (1986) reported that the distribution of porphyrins is directly associated with the number of LDL receptors in those tissues (liver > kidney > lung > spleen) and this was partially supported by the present results: liver levels were greater than kidney concentrations for 12 of the 13 porphyrins; kidney > lung for nine of the 13; lung > spleen for five of the 13. Tumour cells have an elevated number of low density lipoprotein receptors (Maziere *et al.*, 1990) and this may account in part for the specific uptake of certain porphyrins into brain tumour compared to normal brain.

*Structure-localisation relationship*

Porphyrins 11 and 12 possess one side group which is identical (morpholino) whereas the other side group represents opposite charge (anionic *vs* cationic). Both porphyrins exhibit a partition coefficient of approximately one, and both are good tumour localisers. This suggests that the morpholino side group may have some role in defining the overall partition coefficient, and hence the resultant tumour localisation. It should also be noted that both of these porphyrins localise to discrete subcellular organelles, and as a consequence may exhibit more precise targeting of photosensitive sites. This may account for their enhanced phototoxicity when com-

**Figure 4** Relationship between tissue distribution and partition coefficient (pH 7.4) for porphyrins with nett cationic side chains (porphyrins 5, 6, 8, 10 and 12). Correlation coefficients were 0.95 (tumour), 0.91 (spleen), 0.81 (liver), 0.92 (lung) and 0.77 (blood).

pared to similar anionic porphyrins (Woodburn *et al.*, 1991). Both of these porphyrins therefore possess the desired dual characteristics of exhibiting good tumour localisation and targeting crucial subcellular organelles, and therefore offer good prospects as lead compounds for evaluation of other monomeric porphyrins for use in PDT.

With respect to the localising ability of other porphyrins, the tumour:skin ratio of 20:1 for uroporphyrin I suggests that this compound may be a good candidate for PDT when compared to a ratio of 2.5:1 (El-Far & Pimstone, 1986) and 1.5:1 (Table I) for HpD. However, uroporphyrin I was found to be a poor photosensitiser both *in vitro* and *in vivo* (Nelson *et al.*, 1990). Good tumour localisation was exhibited by 11 and 12 with tumour:skin ratios of 7.2:1 and 2.5:1 respectively and these porphyrins should therefore be tested for PDT activity *in vivo*.

The reasons, or any theories, regarding the mechanism of uptake of these porphyrins into the various tissues studied cannot, as yet, be proposed since more extensive phamarcological data needs to be acquired. Since the effectiveness of any photosensitiser depends on its circulation and distribution properties, the interaction of the photosensitiser with serum proteins (and in particular lipoproteins) should be considered. The distribution of porphyrins among proteins is generally thought to be dependent on chemical structure (Jori, 1989) and it is for this reason that the tissue distributions of porphyrins is expected to be dependent upon their hydrophobicity, and hence upon their partition coefficient.

Photosensitisers which exhibited an increase of lipophilicity with decreasing pH have been shown to be retained more in tumour tissue than those compounds which exhibit a different trend (Moan *et al.*, 1987), and this is consistent with the tissue distributions observed in the present study (Figure 2a). HpD (1) and porphyrin 11 had significant concentration levels in tumour and their pH profiles showed an increasing lipophilicity with decreasing pH. Porphyrin 8 had negligible concentration levels in tumour and exhibited a decrease in lipophilicity with decreasing pH. The acidity of tumour tissue (Thistlethewaite *et al.*, 1985) is thought to render membranes more porphyrin soluble (Thomas & Girotti, 1989 and Pottier, 1990) thus the establishment of the pH dependence of partition coefficient may be an important aid in viewing the effectiveness of compounds to localise in tumours.

Based on the limited data set of porphyrins studied in this work, there appeared to be a complementary nature of tissue localisation between the cationic and anionic porphyrins used in this study. At 24 h the anionic porphyrins displayed minimal tissue uptake at $\pi$ of approximately zero whereas the cationic porphyrins displayed maximal tissue uptake at similar $\pi$ values.

### *Use in PDT*

Although high concentrations of the porphyrins exist in liver, kidney and spleen, the monomeric porphyrins 2–13 are still good candidates for use in PDT, especially in relation to intracranial tumours where the porphyrin is administered prior to surgical debulking, and then the remaining tumour is

**Figure 5** Relationship between tissue distribution and partition coefficient (pH 7.4) for porphyrins with nett anionic side chains (porphyrins 2, 4, 7, 9 and 13). Correlation coefficients were 0.74 (tumour), 0.64 (skin), 0.98 (muscle), 0.85 (kidney), 0.73 (spleen), 0.80 (liver), 0.72 (lungs) and 0.82 (blood).

irradiated with laser – in this instance there is little effect of porphyrin localisation on other tissues since these tissues are not directly irradiated and the only disadvantage is the requirement for the patient to avoid sunlight for some time after treatment (Kaye, 1989). Brain tissue is one of the most transparent tissues (Muller & Wilson, 1987) and effective tumour kill is therefore enhanced compared to other tissues which display less light transparency.

### *Effect of subcellular localisation*

The *in vitro* subcellular localisation sites of porphyrins 1–13 have previously been studied using confocal laser scanning microscopy (Woodburn *et al.*, 1991). It was generally found that those porphyrins with overall cationic pendant side chains were retained by the mitochondria whereas those with a dominant anionic character localised in lysosomes. Intracellular localisation sites are important when considering the effects of phototoxicity (Woodburn *et al.*, in press). The porphyrins that localised in the mitochondria were the most phototoxic. Those that localised in lysosomes were also phototoxic and provide a good target site in *in vivo* situations since photoinduced destruction may lead to release of hydrolytic enzymes and subsequent surrounding cell death. Porphyrins 11 and 12 showed good tumour localising capacities at 24 h post injection compared to the surrounding normal brain and are therefore good candidates for further investigation for PDT of brain tumours. Both porphyrins were found to be more phototoxic than HpD in an *in vitro* model due to their possible targeting of lysosomes and mitochondria localisation followed by uptake into lysosomes. The question arises as to what is responsible for ultimate cell and tissue death: concentration effects (porphyrin localising capacity) or the targeting of specific intracellular localisation sites. *In vitro* photonecrosis experiments are currently in progress to answer this fundamental question.

### Conclusions

The best lead compounds for further studies of tumour localising monomeric porphyrins for use in PDT are porphyrins 12 and (to a lesser extent) 11. Not only do these compounds exhibit good tumour:normal brain distributions with the present tumour model, they also possess highly desirable subcellular localisation properties (11, lysosomes; 12, mitochondria then lysosomal engorgement), with greater predicted phototoxicity than comparable anionic porphyrins.

### References

BOWN, S.G. (1990). Photodynamic therapy to scientists and clinicians – one world or two? *J. Photochem. Photobiol. B.*, **6**, 1.

BYRNE, C.J., MARSHALLSAY, L.V. & WARD, A.D. (1987). The structure of the active material in haematoporphyrin derivative. *Photochem. Photobiol.*, **46**, 575.

DOUGHERTY, T.J. (1987). Studies of the structure of porphyrins contained in Photofrin II. *Photochem. Photobiol.*, **46**, 569.

EL-FAR, M. & PIMSTONE, N. (1986). Selective *in vivo* tumour localisation of uroporphyrin isomer I in mouse mammary carcinoma: superiority over other porphyrins. *Cancer Res.*, **46**, 4390.

HENDERSON, B.W. & BELLNIER, D.A. (1989). Tissue localization of photosensitizers and the mechanism of photodynamic tissue destruction. In *Photosensitising Compounds: Their Chemistry, Biology and Clinical Use.* Bock, G. & Harnett, S. (eds) p. 112. Ciba Foundation Symposium 146, Wiley: Chichester.

FORBES, I.J., COWLED, P.A., LEONG, A.S.Y. & 4 others (1980). Phototherapy of human tumours using hematoporphyrin derivative. *Med. J. Aust.*, **2**, 489.

GOMER, C.J. & DOUGHERY, T.J. (1979). Determination of [3H]- and (14C)haematoporphyrin derivative in malignant and normal tissue. *Cancer Res.*, **39**, 146.

HILL, S.H., KAYE, A.H., SAWYER, W.H., MORSTYN, G., MEGISON, P.D. & STYLLI, S.S. (1990). Selective uptake of hematoporphyrin derivate into human cerebral glioma. *Neurosurg.*, **26**, 248.

JORI, G. (1989). *In vivo* transport and pharmacokinetic behaviour of tumour photosensitisers. In *Photosensitising Compounds: Their Chemistry, Biology and Clinical Use.* Bock, G. & Harnett, S. (eds) p. 78. Ciba Foundation Symposium 146, Wiley: Chichester.

JORI, G. & REDDI, E. (1990). Strategies for tumour targeting by photodynamic sensitisers. In *Photodynamic Therapy of Neoplastic Diseases*, Kessel, D. (ed.) Vol II, p. 117. CRC Press, Boca Raton.

KAYE, A.H., MORSTYN, G. & ASHCROFT, R.G. (1985). Uptake and retention of haematoporphyrin derivative in an in vivo in vitro model of cerebral glioma. *Neurosurg.*, **17**, 883.

KAYE, A.H., MORSTYN, G., GARDNER, I. & PYKE, K. (1986). Development of a xenograph glioma model in mouse brain. *Cancer Res.*, **46**, 1367.

KAYE, A.H., MORSTYN, G. & APUZZO, M.L.J. (1988). Photoradiation therapy and its potential in the management of neurological tumours. *J. Neurosurg.*, **69**, 1.

KAYE, A.H. (1989). Photoradiation therapy of brain tumours. In *Photosensitising Compounds: Their Chemistry, Biology and Clinical Use.* Bock, G. & Harnett, S. (eds) p. 209. Ciba Foundation Symposium 146, Wiley: Chichester.

KESSEL, D. (1977). Effects of photoactivated porphyrins at the cell surface of leukemia L1210 cells. *Biochem.*, **16**, 3443.

KESSEL, D. (1981). Transport and binding of haematoporphyrin derivatives and related porphyrins by murine leukemia L1210 cells. *Cancer Res.*, **41**, 1318.

KESSEL, D. & CHENG, M.L. (1985). Biological and biophysical properties of the tumour localising component of haematoporphyrin. *Cancer Res.*, **45**, 3053.

KESSEL, D. (1986). Porphyrin-lipoprotein association as a factor in porphyrin localisation. *Cancer Lett.*, **33**, 183.

KESSEL, D., THOMPSON, P., MUSSELMAN, B. & CHANG, C.K. (1987). Chemistry of haematoporphyrin-derived photosensitisers. *Photochem. Photobiol.*, **46**, 563.

MARTIN, Y.C. (1981). A practitioner's perspective of the role of quantitative structure-activity analysis in medicinal chemistry. *J. Med. Chem.*, **24**, 229.

MAZIERE, J.C., SANTUS, R., MOLIERE, P. & 7 others (1990). Cellular uptake and photosensitising properties of anticancer porphyrins in cell membranes and low and high density proteins. *J. Photochem. Photobiol. B.*, **6**, 61.

MOAN, J., PENG, Q., EVENSEN, J.F., BERG, K., WESTERN, A. & RIMINGTON, C. (1987). Photosensitising efficiencies, tumour- and cellular uptake of different photosensitising drugs relevant for photodynamic therapy of cancer. *Photochem. Photobiol.*, **46**, 713.

MULLER, P.J. & WILSON, B.D. (1987). Photodynamic therapy of malignant primary brain tumours: clinical effects, post-operative ICP, and light penetration of the brain. *Photochem. Photobiol.*, **46**, 929.

NELSON, J.S., ROBERTS, W.G., LIAW, L. & BERNS, M.W. (1990). Cellular and tumour model studies using several PDT sensitizers. In *Photodynamic Therapy of Neoplastic Diseases*, Kessel, D. (ed.) Vol I, p. 147. CRC Press, Boca Raton.

POTTIER, R.H. (1990). Localisation phenomena: pH effects. In *Photodynamic Therapy of Neoplastic Diseases*, Kessel, D. (ed.) Vol II, p. 63. CRC Press, Boca Raton.

RIMINGTON, C., SOMMER, S. & MOAN, J. (1987). Hematoporphyrin ethers-I. Generalized synthesis and chemical properties. *Int. J. Biochem.*, **19**, 315.

SCOURIDES, P.A., MORSTYN, G. & NGU, M. (1986). An improved synthesis of porphyrin C. *J. Chem. Soc. Chem. Commun.*, **24**, 1817.

SLAMA, J.T., SMITH, H.W., WILSON, C.G. & RAPOPORT, H. (1975). Porphyrin-protein bond of cytochrome C. *J. Amer. Chem. Soc.*, **97**, 6556.

THISTLETHEWAITE, A.J., LEEPER, D.B., MOYLAR III, D.J. & NERLENGER, R.E. (1985). pH distribution in human tumours. *Int. J. Radiat. Oncol. Biol. Phys.*, **11**, 1647.

THOMAS, J.P. & GIROTTI, A.W. (1989). Glucose administration augments in vivo uptake and phototoxicity and the tumour-localising fraction of haematoporphyrin derivative. *Photochem. Photobiol.*, **49**, 241.

WALKER, M.D., GREEN, S.B., BYAR, D.P. & ALEXANDER, E. (1980). Randomised comparisons of radiotherapy and nitrosoureas for the treatment of malignant glioma after surgery. *N. Engl. J. Med.*, **303**, 1324.

WOODBURN, K.W., VARDAXIS, N.J., HILL, J.S., KAYE, A.H. & PHILLIPS, D.R. (1991). Subcellular localisation of porphyrins using confocal laser scanning microscopy. *Photochem. Photobiol.*, **54**, 725.

WOODBURN, K.W., VARDAXIS, N.J., HILL, J.S., KAYE, A.H., REISS, J.A. & PHILLIPS, D.R. (1992). Evaluation of porphyrin characteristics required for photodynamic therapy. *Photochem. Photobiol.* (in press).